# Quantitative distribution of flavan-3-ols, procyanidins, flavonols, flavanone and salicylic acid in five varieties of organic winter dormant Salix spp. by LC-MS/MS

**DOI:** 10.1016/j.heliyon.2024.e25129

**Published:** 2024-02-01

**Authors:** Mihai Victor Curtasu, Natalja P. Nørskov

**Affiliations:** Department of Animal and Veterinary Sciences, Aarhus University, Blichers Alle 20, 8830 Tjele, Denmark

**Keywords:** Salix spp, Salicaceae, willow, Quantification, phenolic compounds, flavonoids, salicylic acid, LC-MS/MS

## Abstract

Willow trees (*Salix* spp.) exhibit remarkable genetic and phenotypic diversity, yielding a broad spectrum of bioactive compounds, notably valuable phenolic compounds such as condensed tannins (phenolic polymers), flavonoids, salicylic glucosides, and phenolic compounds. These enhance the economic value of willow crops and make them suitable for circular bioeconomy. Phenolic compounds known for their diverse applications as antioxidants, antimicrobial agents, pharmaceuticals, nutraceuticals and antiseptics and more, find a natural source in willow. This study aimed to elucidate the composition of 12 flavonoids and salicylic acid in different segments of five organic winter dormant willow species (*S. daphnoides, S. fragilis, S. dasyclados, S. viminalis,* and *S. dasyclados x viminalis*) using quantitative analysis and providing valuable insights into their high-value phenolic compounds. Separation into buds, wood and bark segments allowed for a precise characterization of the location of certain phenolic compounds and quantification using LC-MS/MS techniques. LC-MS/MS is an analytical technique known for its increased sensitivity and chromatographic precision. Among the findings, catechin emerged as the predominant flavan-3-ol in all Salix species, with the highest concentration in the buds of *Salix viminalis* (7.26 mg/g DM). Naringenin exhibited species-specific variations, with S. dasyclados and S. viminalis recording the highest levels. Salicylic acid concentrations peaked in *S. dasyclados* (5.38 mg/g DM) and *S. daphnoides* (4.43 mg/g DM), particularly within the bark. When evaluating other individual flavonoids and total polyphenol content (TPC), disparities between buds, bark, and wood became evident, with wood consistently displaying the lowest content. Notably, the higher concentration of polyphenolic compounds in willow bark can be attributed to its susceptibility to external threats and its role as a robust defense mechanism against pathogens and herbivores. This study underscores the significance of diverse willow species as a source of high-value phenolic compounds, distributed differentially across plant parts and species. This knowledge holds promise for their potential applications in the circular bioeconomy.

## Highlights

• Winter dormant willow contains polyphenolic compounds of valuable interest.
• High concentrations of catechin, naringenin and salicylic acid.
• High variations in individual flavonoids were observed.
• Constantly low concentrations of all measured polyphenolic compounds in wood subparts.
• All five varieties contained high TPC in buds and bark, highest in bark of S. dasyclados x viminalis (6.7 %).

## Graphical abstract

**Figure:**
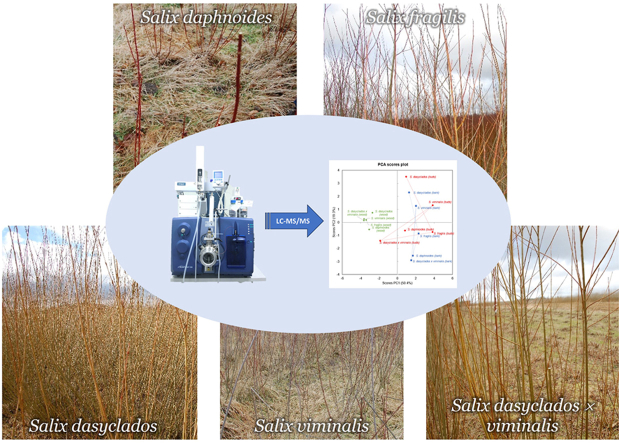

## 1 Introduction

Historically, the willow tree (Salix spp.) has been of interest to humans for thousands of years, and records suggest the first use of willow extract as an analgetic, antipyretic and anti-inflammatory agent dates back to 6000 years ago in Mesopotamia [1,2]. A historical and cultural perspective related to aspirin and other willow derived compounds has been described in more detail by Mahdi et al. (2006) as ancient physicians used herbal extracts to cure pain and inflammation during the Babylonian, Assyrian and Sumerian civilizations [2]. However, not before the 18th century, organic chemists discovered the active compounds of willow, salicin and salicylic acid. When consumed, salicin is degraded by intestinal enzymes and bacteria to saligenin and glucose. Saligenin is further oxidized in the blood and liver to salicylic acid [3]. Salicylic acid has been recognized as the key precursor molecule contributing to the discovery of acetylsalicylic acid, traded as aspirin [2]. Traditionally, salicin has been used as a biomarker for the activity of willow bark. However, recent studies attribute the bioactivity of willow bark not only to the presence of salicin and salicylic acid but to the high content of different phenolic compounds (PCs) [1,4]. Further development in analytical techniques led to the phytochemical profiling of willow bark extracts with discovery of heterogeneous composition of PCs [5]. Compounds identified belong to the different classes of PCs, such as condensed tannins, phenolic polymers, flavonoids, salicylic glucosides and phenolic acids [6]. Besides the traditional basket manufacturing and pharmaceutical value, willow has gained increasing interest due to its short rotation coppice, as a fast-growing tree with high biomass yield from low agricultural inputs [1,7]. Thus, willow has become an important bioenergy crop in temperate regions [7]. This combination of having a high content of high-value compounds with high biomass yield per hectare and different industrial applications makes willow an attractive crop for circular bioeconomy. The prospect of combining biorefining of high-value compounds for multidirectional products with bioenergy utilization could become a sustainable solution. Its short rotation coppice system allows for regular harvests of biomass, which can be used for various purposes, including bioenergy production. The combination of high-value compounds and biomass production aligns with the principles of a circular bioeconomy. In a circular bioeconomy, resources are efficiently used, waste is minimized, and the value of agricultural products is maximized. Willow's ability to provide both valuable compounds and biomass contributes to the sustainability of this approach. In terms of green transition, there is also an advantage to finding green alternatives to synthetic compounds used in pharmaceutical and chemical industries, reducing the environmental impact of various industries [7]. Furthermore, the fast growth of willow trees contributes to carbon sequestration, helping mitigate the effects of climate change. Additionally, the use of willow biomass for bioenergy reduces reliance on fossil fuels, further enhancing its environmental benefits.

Willow includes approximately 430–440 species and an unknown number of natural and artificial hybrids [5]. Because there is a high number of species, an effort has been made to identify the willow species that combine high content of PCs with high willow biomass yield per hectare. Therefore, a lot of gaps in knowledge exist even for most of the common willow species, but moreover for unknown or uncharacterized willow species. Another important aspect is the lack of detailed quantitative data on individual high-value phenolic compounds and distribution in plant parts. In the study of Brereton et al. (2017), three willow species were investigated for the highest PCs content per hectare per year, *Salix dacyclados, Salix viminalis* and *Salix miyabeana* [7]. *Salix miyabiana* has produced the highest phenolic and condensed tannin yield. However, the PC yield also highly depended on growth location. Another study investigated ten willow genotypes cultivated annually for the highest salicin yield per hectare [8]. The highest salicin yield (over 92 kg/ha) has been obtained for *Salix purpurea* x *Salix daphnoides* hybrids [8]. Other studies have focused on the distribution and identification of PCs in willow buds, bark, wood, and leaves. In the study of Lavola et al. (2018), it was shown that buds and bark contained a high concentration of PCs compared to wood [6]. Bark was generally higher in PCs, especially in salicylate glycosides and simple phenolic glucosides. Condensed tannins and flavonoid glucosides showed similarity in content between buds and bark [6]. In general, the content of PCs was relatively similar between different-aged trees [6]. Metabolomics study on willow bark and leaves identified 29 and 34 PCs in *Salix alba*'s leaves and bark, respectively, belonging to derivatives of phenolic acids, flavan-3-ols, flavonols and procyanidins [9]. They estimated the total PC content to be 5595.96 and 2330.31 mg/100 g of dry weight in leaves and bark, respectively [9]. Growth conditions, including climate, soil quality, and geographical location, strongly influence the phenolic compound yield in willow [7]. Factors such as temperature, precipitation, and soil nutrients can impact the plant's metabolic pathways and its production of phenolic compounds [7,10,11]. Furthermore, selection of specific genetic lines or cultivars can be crucial for optimizing the phenolic compound yield. In some cases, hybridization or selective breeding programs may be employed to develop willow varieties with enhanced phenolic compound production [7,11,12].

Flavonoids are the primary group of PCs and by far the most diverse group, counting around 7000 identified compounds [13,14]. Plants produce flavonoids to defend against pathogenic fungi and bacteria, herbivorous insects or reactive oxygen species (ROS) [14]. The chemical structure of all flavonoids is based on the hydrocarbon skeleton of flavone and two biosynthetic pathways generate flavonoid-based compounds, shikimic acid and acetate pathways [13,14]. Individual flavonoid compound differs through the different substituents to the benzene rings by hydroxylation, methylation, acylation and glycosylation with mono- or oligosaccharides [14].

Although several studies explored the total PC content of willow in terms of yield, plant part or phytochemical fingerprint, studies on the detailed quantitative composition of individual high-value PC in different willow species are lacking. The aim of this study was, therefore, to perform comprehensive quantitative analyses of 12 flavonoids and salicylic acid in the buds, bark, and wood of five willow species, *Salix daphnoides, Salix fragilis, Salix dasyclados, Salix viminalis* and *Salix dasyclados x viminalis*.

## 2 Results and discussion

### 2.1 Monomeric and polymeric flavan-3-ols

**Fig. 1:**
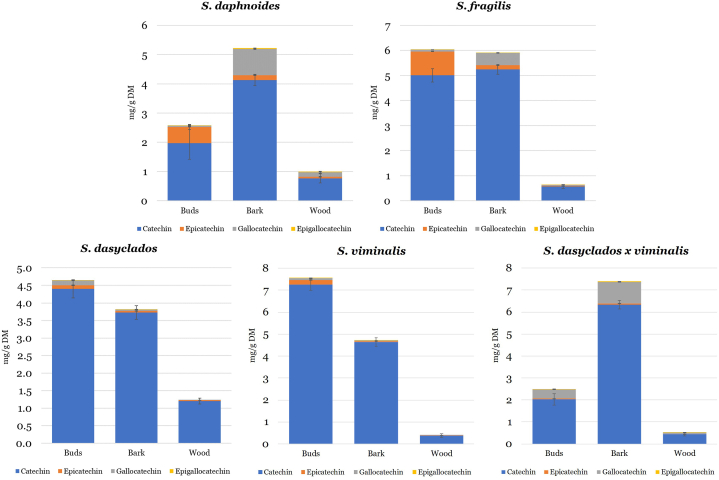
The concentration (mg/g DM) and distribution of catechin, epicatechin, gallocatechin, and epigallocatechin in five dormant varieties of willow and distribution between buds, bark, and wood.

Plants can produce a plethora of different flavonoids, and particular families of plants tend to specialize in specific flavonoids for their defense [15]. Willow is known to produce high quantities of flavonoids known as flavan-3-ols of monomeric and polymeric structures [8]. The concentration (mg/g DM) and distribution of monomeric flavan-3-ols, catechin, epicatechin, gallocatechin and epigallocatechin in buds, bark and wood of *Salix daphnoides, Salix fragilis, Salix dasyclados, Salix viminalis,* and *Salix dasyclados x viminalis* are shown in Fig. 1.

Catechin was by far the most dominant flavan-3-ol in all *Salix* species, with the highest concentration of 7.26 mg/g DM in the buds of *Salix viminalis*, Table 1. It has been previously reported that the content of catechin can constitute the major part (81 %) of monomeric and polymeric flavan-3-ols [16], which can indicate that catechin is the main flavan-3-ol in willow. As expected, wood contained the lowest concentration of catechin (0.39–1.21 mg/g DM) and, in general, other flavan-3-ols compared to buds and bark, similar results were reported previously [6]. Buds and bark of the willow showed high variability in the concentration of flavan-3-ols ranging between 2.5 and 7.56 mg/g DM in the buds and 3.8–7.41 mg/g DM in the bark. In two species, *Salix daphnoides* and *Salix dasyclados x viminalis* buds contained significantly lower concentrations of flavan-3-ols. Oppositely to *Salix viminalis, Salix dasyclados* and *Salix fragilis,* the concentration of flavan-3-ols was significantly higher. The isomeric form of catechin, epicatechin, was mainly present in the buds of *Salix daphnoides* and *Salix fragilis*, whereas gallocatechin was mainly present in the bark of *Salix daphnoides, Salix fragilis* and *Salix dasyclados x viminalis*. Only trace amounts of epigallocatechin, the isomeric form of gallocatechin, were detected in the buds and bark of all five *Salix* species. A higher fluctuation in the concentration of catechin and flavan-3-ols generally in buds and bark of willow species was observed. However, the bark is the plant part most extensively investigated previously. In our study, the concentration of catechin reached 6.34 mg/g DM in the bark of *Salix dasyclados x viminalis*. Although some fluctuation in the catechin concentration has been observed among willow species, the catechin concentration was generally high, ranging between 3.73 and 6.34 mg/g DM. Previous reports indicate similar concentrations in the bark of willow, 0.5 % has been reported in the *Salix daphnoides* [3]. The concentration of catechin in willow bark of different willow clones has been investigated in the study of Poblocka-Olech et al. (2007), in which concentrations ranged between 71.55 and 427.55 mg/100 g [17]. Bark was collected in March [17] and therefore is comparable to our study. Slightly higher concentrations of catechin have been reported in the buds and bark of winter dormant *Salix pyrolifolia* ranging between 6.36 and 11.27 mg/g DW depending on the part and age of willow trees [6]. To our knowledge, no previous studies on the content of epicatechin and gallocatechin have been reported. The concentration of epicatechin reached 0.96 g/mg DM in the buds of *Salix fragilis* and gallocatechin 0.98 g/mg DM in the bark of *Salix dasyclados x viminalis*, indicating low content compared to catechin*.* The reports on the presence of catechin and gallocatechin in the bark of willow date back to 1968–1969 [18]. Catechin and flavan-3-ols have been proven to be strong anti-oxidants [19]. In plant tissue, catechin and epicatechin provide resistance against fungal attack and prevent the proliferation of neighboring plant species [20]. The catechin concentration has been shown to change in response to stress, such as water deficiency [10]. The general perception of the role of flavan-3-ols in plants is related to their protection against harmful intruders such as bacteria, fungi, insects and herbivorous animals [20]. Metabolomics profiling studies have also revealed catechin, epicatechin and gallocatechin as the main flavan-3-ols detected in the bark and leaves of willow [9,21]. Similarly, the results in this study show that in all five species of willow catechin is the main compound from the class of flavan-3-ols.

**Table 1:** Concentrations (mg/g of dry matter (DM)) of the individual flavan-3-ol, polymeric flavan-3-ols, flavonols and flavonol glucosides, flavanone (naringenin) and salicylate (salicylic acid), measured in five varieties of willow and distribution between buds, bark, and wood (results are expressed as LSmeans ± SEM).

Compound	Part	*S. daphnoides*	*S. fragilis*	*S. dasyclados*	*S. viminalis*	*S. dasyclados* x *viminalis*	SEM	P-value
Monomeric flavan-3-ols								
Catechin	Buds	1.97c	5.01b	4.4b	7.26a	2.03c	0.27	0.0001
	Bark	4.14dc	5.24b	3.73d	4.64bc	6.34a	0.19	<0.0001
	Wood	0.77b	0.58bc	1.21a	0.39c	0.45c	0.06	<0.0001
Epicatechin	Buds	0.56b	0.96a	0.11cd	0.2c	0.04d	0.03	<0.0001
	Bark	0.17a	0.17a	0.05a	0.05a	0.07a	0.04	0.0865
	Wood	0.07a	0.03b	0.03b	0.01c	0.01c	0.01	<0.0001
Gallocatechin	Buds	0.05c	0.07c	0.14b	0.1bc	0.43a	0.01	<0.0001
	Bark	0.9b	0.51c	0.04d	0.03d	0.98a	0.02	<0.0001
	Wood	0.16a	0.04bc	0.01c	0.01c	0.07b	0.01	<0.0001
Epigallocatechin	Buds	0.003b	0.004b	0.006b	0.005b	0.016a	0.002	0.0019
	Bark	0.033a	0.017c	0.002d	0.001d	0.027b	0.001	<0.0001
	Wood	0.016a	0.004b	–	–	0.002b	0.002	0.0024
SUM F3OL	Buds	2.58c	6.03ab	4.65b	7.56a	2.5c	0.30	0.0002
	Bark	5.22bc	5.92b	3.82d	4.71cd	7.41a	0.21	<0.0001
	Wood	1.0a	0.65b	1.24a	0.41b	0.53b	0.07	<0.0001
**Polymeric flavan-3-ols**								
Procyanidin B1	Buds	0.5b	1.04a	0.71b	1.27a	0.13c	0.06	0.0002
	Bark	0.67b	1.01a	0.88a	0.96a	0.52b	0.05	<0.0001
	Wood	0.1b	0.07bc	0.19a	0.03cd	0.02d	0.01	<0.0001
Procyanidin B2	Buds	0.38a	0.51a	0.05b	0.09b	0.02b	0.03	0.0001
	Bark	0.09a	0.09a	0.02a	0.02a	0.02a	0.02	0.0363
	Wood	0.03a	0.01b	0.01bc	0.01c	0.01bc	0.01	<0.0001
Procyanidin C1	Buds	0.5b	1.1a	0.54b	0.98^a^	0.12b	0.08	0.0015
	Bark	0.5c	1.03a	0.85 ab	0.83b	0.63c	0.05	<0.0001
	Wood	0.06b	0.05b	0.12a	0.01c	0.01c	0.01	<0.0001
SUM CT	Buds	1.38b	2.64a	1.29b	2.34a	0.26c	0.16	0.0006
	Bark	1.25c	2.12a	1.75b	1.8ab	1.16c	0.08	<0.0001
	Wood	0.18b	0.12b	0.31a	0.04c	0.03c	0.02	<0.0001
**Flavonols**								
Quercetin	Buds	0.09a	0.09a	0.04a	0.06a	0.08a	0.010	0.062
	Bark	0.12a	0.08b	0.05c	0.12a	0.12a	0.006	<0.0001
	Wood	0.01a	0.01a	0.01a	0.01a	0.01a	0.001	0.097
Kaempferol	Buds	0.021a	0.022a	0.022a	0.026a	0.019a	0.002	0.424
	Bark	–	–	–	–	–		
	Wood	–	–	–	–	–		
**Flavonol glucoside**								
Rutin	Buds	0.33a	0.19b	0.01c	0.22b	0.05c	0.016	0.0002
	Bark	0.13a	0.06b	0.04c	0.12a	0.01d	0.002	<0.0001
	Wood	0.01a	0.01b	0.01b	0.01b	0.01b	0.0002	<0.0001
**Flavanone**								
Naringenin	Buds	0.04c	0.03c	6.72a	2.34b	0.03c	0.27	<0.0001
	Bark	0.07c	0.07c	2.83a	1.97b	0.05c	0.09	<0.0001
	Wood	0.01c	0.01c	1.2a	0.43b	0.01c	0.03	<0.0001
**Salicylate**								
Salicylic acid	Buds	1.13b	1.09b	2.15 ab	3.52a	0.23b	0.36	0.008
	Bark	4.43a	2.22b	5.38a	3.97a	0.18c	0.33	<.0001
	Wood	0.65b	0.5b	0.96a	0.02c	–	0.04	<.0001

^∗^different superscripts letters (a-d) denote significant differences between comparisons.

**Fig. 2:**
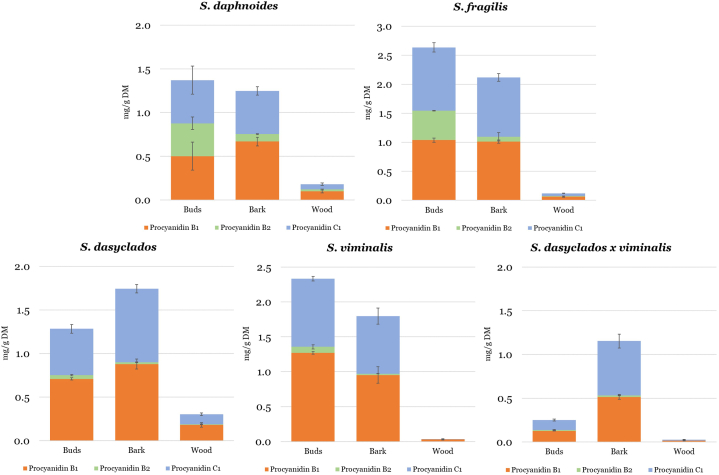
The concentration (mg/g DM) and distribution of polymeric flavan-3-ols (Procyanidin B1, procyanidin B2, and procyanidin C1) in five dormant varieties of willow and distribution between buds, bark, and wood.

Another group of compounds in the flavan-3-ols class is their polymeric derivatives, procyanidins. Procyanidins are polymers of monomeric units of catechin, epicatechin, and prodelphinidins are polymers of gallocatechin and epigallocatechin. These polymeric structures are often referred to as polymeric flavan-3-ols, vegetable tannins, polyflavans, and other terms, but should not be confused with hydrolysable or complex tannins of high molecular weight that have the capacity to precipitate proteins [20,22,23]. The concentration (mg/g DM) and distribution of procyanidins, procyanidin B1 and B2 and procyanidin C1 in buds, bark, and wood of *Salix daphnoides, Salix fragilis, Salix dasyclados, Salix viminalis* and *Salix dasyclados x viminalis* are shown in Fig. 2.

The concentration of procyanidins was generally lower than their monomeric derivative, with the highest concentration of all three procyanidins found in buds of *Salix fragilis* 2.63 mg/g DM (Table 1). Procyanidin B1 and procyanidin C1 were found in higher concentrations than procyanidin B2. A fluctuation in the concentrations between buds and bark was again observed; however, the concentration of procyanidins in wood was constantly low for all five willow species. The highest procyanidin B1 and C1 concentrations were detected in the *Salix fragilis* bud*s* and *Salix viminalis*. Buds of *Salix fragilis* and *Salix daphnoides* contained the highest concentrations of procyanidin B2. Metabolomics profiling studies indicate as well the presence of other procyanidin types [9]. In the study of Piatczak et al. (2020), A-type and B-type, mainly as dimers and trimers, were tentatively identified in the bark and leaves of *Salix alba* [9]. However, no procyanidin A2 was detected in the five willow species in this study. When screening for dimeric procyanidins in the bark of *Salix alba,* Esatbeyoglu et al. (2010) have identified procyanidin B1, B3, B6 and B7 [16]. Most published articles on the concentration of procyanidins in willow report concentrations as the total concentration of condensed tannins, lacking detailed knowledge about concentrations of individual procyanidins. That can be explained by the challenges in the analytical methods of these compounds such as the lack of standards for quantification on LC-MS/MS. However, analyzing procyanidins as the total concentration of condensed tannins using spectrophotometric methods can lead to an overestimation of the content due to interferences [24]. Nevertheless, these compounds are essential for willow survival as they play a direct role in the defense mechanisms [15,23]. Condensed tannins are considered deleterious to phytophagous insects, a deterrent to herbivore feeding and important anti-microbial compounds [15,23]. Their industrial use has also been described extensively in the literature with importance to the leather industry as tanning agents, wood adhesives, anti-corrosive primers, and food and feed additives [24]. They positively impact human health and benefit ruminant livestock by improving nitrogen nutrition and providing protection from pasture bloat [23,25]. Therefore, further development in analytical chemistry and analytical methods is important to shed light on the concentrations of individual procyanidin in willow species. The highest yield on the total concentration of condensed tannins has been estimated to be 35.54 kg/ha/year for one of the willow cultivars in the study of Brereton et al. (2017) [7]. However, high variation among the willow cultivars and growth location has been observed [7].

### 2.2 Flavonols

**Fig. 3:**
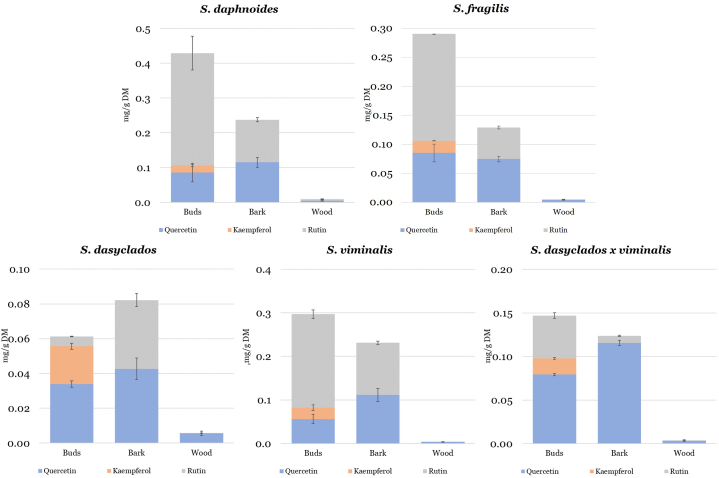
The concentration (mg/g DM) and distribution of quercetin, kaempferol, and rutin in five dormant varieties of willow and distribution between buds, bark, and wood.

The concentration (mg/g DM) and distribution of flavonols, quercetin and kaempferol and flavonol glucoside, rutin in buds, bark, and wood of *Salix daphnoides, Salix fragilis, Salix dasyclados, Salix viminalis* and *Salix dasyclados x viminalis* are shown in Fig. 3. The concentration of quercetin was highest in the bark of *Salix daphnoides, Salix viminalis* and *Salix dasyclados x viminalis,* 0.12 mg/g DM, followed by *Salix fragilis* with the lowest concentration in the bark of *Salix dasyclados* (Table 1). There was no variation in the concentration of quercetin and kaempferol in the buds of all five willow species. Kaempferol was only quantifiable in the buds. In the study of Budny et al. (2021), the concentration of quercetin varied between 0.00057 and 0.59 mg/g DM, and kaempferol varied between 0.00012 and 0.00247 mg/g DM in the young shoots of different willow cultivar collected in May [5]. However, because bark and wood were not separated, the results are not directly comparable to our study. Higher fluctuation in the concentrations of rutin has been observed for the buds and bark of five willow species, with the highest concentration in the buds of *Salix daphnoides* and the bark of *Salix daphnoides* and *Salix viminalis*. The wood contained the lowest concentration of quercetin, kaempferol and rutin, with no difference between willow species. To our knowledge, no other studies report the content of quercetin, kaempferol and rutin in buds, bark, and wood of winter dormant Salix spp.

Several metabolomic profiling studies report a high number of quercetin and kaempferol glucosides derivatives [6,9,21]. In the study of Piatczak et al. (2020), ten different quercetin glucoside derivatives were identified [9]. In the studies of Lavola et al. (2018) quercetin 3-*O*-galactoside, quercetin 3-*O*-glucoside and kaempferol 3-*O*-glucoside were identified [6]. These studies indicate that quercetin is mainly present as glucoside in willow. That was also the case for rutin (quercetin 3-*O*-rutinoside), whose concentration was generally higher than quercetin aglycon for *Salix daphnoides, Salix viminalis* and *Salix fragilis*. The challenge of quantifying such a high number of different glucosides is the requirement for their corresponding analytical standards. In some studies, these compounds have been referred to as flavonoids glycosides and quantified as total glucosides [6,26]. In another study, hydrolysis of quercetin glucosides was performed to quantify quercetin as its aglycon in the bark of ten *Salix purpurea* genotypes [8] and the mean concentration of quercetin in the willow bark was measured at 0.11 mg/g DM, which is comparable to our study. In the study of Piatczak et al. (2020), the content of flavonols, based on the flavonol glucosides has been estimated to 29.59 mg/100 g DW (0.23 mg/g DW) in the bark of *Salix alba*. Differences in the results reflect the challenges in the analytical procedures to measure these compounds in willow, requiring further development in analytical techniques.

In plants, glucosidation can have different functionalities such as detoxification, stabilization and increased hydrophilicity [13,15,23]. Quercetin has been identified as a multi-functional compound in plants. Quercetin is an allelochemical, anti-fungal compound, also an important pigment serving as a visual signal for attracting pollinators and a signaling compound in plant-insect interactions [15]. Further, quercetin has been reported to be a beneficial compound for human health with anti-inflammatory, anti-oxidant, anti-microbial and other beneficial effects [27]. Warminski et al. (2021) have estimated the yield of quercetin to be 0.114–0.905 kg/ha, though high variation in the concentration of quercetin among the willow genotypes has been observed [8]. Brereton et al. (2017) have estimated the yield of flavonols to be 0.97–1.45 kg/ha/year with high variation among willow cultivars and growth site [7]. Further research on the quantification and the yield of these high-value compounds in willow biomass is warranted.

### 2.3 Naringenin

**Fig. 4:**
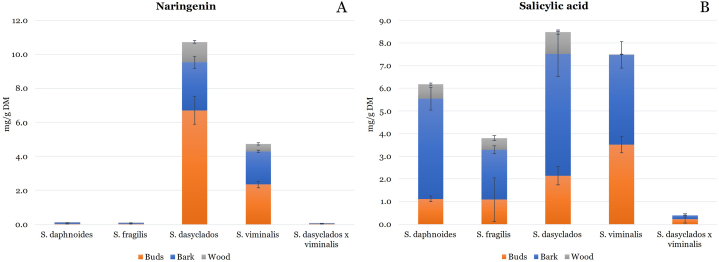
The concentration (mg/g DM) and distribution of naringenin (A) and salicylic acid (B) in buds, bark, and wood from five dormant varieties of willow.

The concentration (mg/g DM) and distribution of flavanone, naringenin in buds, bark and wood of *Salix daphnoides, Salix fragilis, Salix dasyclados, Salix viminalis* and *Salix dasyclados x viminalis* are shown in Fig. 4A. The concentration of naringenin was highest in *Salix dasyclados* in all plant parts, buds 6.72 mg/g DM, bark 2.83 mg/g DM and wood 1.2 mg/g DM. The second highest concentration of naringenin was measured in *Salix viminalis*, buds 2.34 mg/g DM, bark 1.97 mg/g DM and wood 0.43 mg/g DM. In contrast, the concentration of naringenin in *Salix daphnoides, Salix fragilis,* and *Salix dasyclados x viminalis* was negligible compared to two other willow species (Table 1). This indicates that the concentration of naringenin is species-specific, which is the opposite of catechin. The concentration of naringenin in *Salix dasyclados* was comparable to the level of catechin measured in five willow species. There was no fluctuation in the concentration between buds and bark as has been observed for flavan-3-ols, procyanidins and flavonols. Higher concentration has been measured in buds, followed by bark and wood in *Salix dasyclados* and *Salix viminalis*. In the study of Lavola et al. (2018), a low concentration of naringenin was measured in the buds of winter-dormant *Salix pyrolifolia*. In contrast, no naringenin has been detected in the bark and wood [6]. Similarly, low concentration of naringenin has been measured in the young shoots of different varieties and cultivars of willow [5]. In our study, the content of naringenin in buds of *Salix dasyclados* and *Salix viminalis* was more than 200 and 40 times higher, respectively, compared to *Salix daphnoides, Salix fragilis* and *Salix dasyclados x viminalis*. A similar pattern was observed for bark and wood. When *Salix daphnoides,* and *Salix purpurea* were drought-stressed, a compound-specific response of flavonoids was observed, specifically for naringenin derivatives [10]. Our study indicates that naringenin can be an important biomarker for specific willow species and environmental stress exposure; however, further research is needed to explore this hypothesis. Three derivatives of naringenin glucoside have been identified and quantified in buds, bark and wood [6]. In Ramos et al., 2019 study, two glucosides of naringenin have been identified, indicating that glucosidation is also an important part of naringenin metabolism in willow [21].

### 2.4 Salicylic acid

The concentration (mg/g DM) and distribution of major salicylate, salicylic acid in buds, bark, and wood of *Salix daphnoides, Salix fragilis, Salix viminalis, Salix dasyclados* and *Salix dasyclados x viminalis* is shown in Fig. 4B. Besides salicin, salicylic acid is the most studied phenolic compound in willow. Generally, the concentration of salicylic acid was highest in the bark compared to buds and lowest in wood. *Salix dasyclados x viminalis*'s bark contained the lowest salicylic acid concentration (Table 1). The highest concentrations have been observed for *Salix dasyclados* and *Salix daphnoides* at 5.38 and 4.43 mg/g DM, respectively, reaching approximately 0.5 %. The lowest concentration of salicylic acid was measured in the bark of *Salix dasyclados x viminalis*, which was significantly lower compared to four other species and can indicate some species specificity. The mean concentration of salicylic acid in willow bark of different genotypes has been measured at 1.37 mg/g DM with high variation among the genotypes 0.11–3.54 mg/g DM [8], comparable to our study. Warminski et al. (2021) observed that the concentration of salicin was systematically higher app. ten times in relation to the concentration of salicylic acid [8]. Over the years, salicylic acid and salicin have been associated with positive health effects for humans and animals, such as anti-inflammatory, anti-rheumatic, antipyretic and analgesic [8,28,29]. Warminski et al. (2021) estimated the yield of high-value compounds such as salicylic acid and salicin to be 11.7–0.28 kg/ha and over 92 kg/ha, respectively, although high variation in the concentrations of salicylic acid and salicin has been observed [8]. The analysis of the salicylic acid is mainly challenged by the glucosidation of salicylic acid, which requires hydrolysis to liberate and analyze salicylic acid as its aglycon. However, other reports of the salicylic acid concentration in willow bark can be found [21]. Given the importance of salicylic acid for pharmaceutical sector and other industrial applications, we focused on the characterization of the hydrolyzed form of salicylic acid, a step employed in several technological processes for separation, purification and concentration of valuable compounds. However, is important to acknowledge the natural forms of salicylic acid as various types of salicylates (e.g., salicin, saligenin, salicortin, isosalicin, picenin, tremulacin, etc.) which play an important role in plan metabolism and defense. These compounds have been reported in previous studies using semi-targeted or untargeted techniques such as Sulima P. et al. (2017) or Kammerer B. et al. (2005) as well as covered in the review by Tyśkiewicz, K. et al. (2019) [[30], [31], [32]].

### 2.5 Total concentration of polyphenolic compounds (TPC)

The total concentration (mg/g DM) of PCs in buds, bark, and wood of *S. daphnoides, S. fragilis, S. viminalis, S. dasyclados,* and *S. dasyclados x viminalis* is shown in Table 2. The highest concentration of PCs was measured in the bark of *S. dasyclados x viminalis,* reaching 66.6 EqG/g DM, whereas the lowest measured for *S. dasyclados* and *S. viminalis* was 37.8 and 37.2 mg EqG/g DM, respectively. The opposite situation was observed for buds with the highest concentration of PCs in the buds of *S. viminalis* 51.9 EqG/g DM and the lowest for *S. dasyclados x viminalis* 29.9 EqG/g DM. This indicates an overall high TPC for all five willow species. The wood contained the lowest concentration of TPC, in agreement with the results from LC-MS quantification and results from the literature [6]. The results of Lavola et al. (2018) showed similar trends of lower contents of salicylate glycosides, simple phenolic glucosides, flavonoid glucosides, phenolic acid, condensed tannins, and other phenolic compounds in wood compared to bark and buds [6], though with high variation between buds and bark and age of the trees depending on the class of PCs [6].

**Table 2:** Total polyphenol content (TPC) measured in five varieties of willow (LSmeans expressed as mg equiv. gallic acid/g DM).

Part	*S. daphnoides*	*S. fragilis*	*S. dasyclados*	*S. viminalis*	*S. dasyclados* x *viminalis*	SEM	P-value
Buds	42.8b	50.8ab	49 ab	51.9a	29.9c	1.6	0.0008
Bark	56b	48.1c	37.8d	37.2d	66.6a	0.8	<0.0001
Wood	12.3a	8.4b	11.8a	6.4c	7.0c	0.2	<0.0001

### 2.6 Principal component analysis (PCA) of *S. daphnoides, S. fragilis, S. viminalis, S. dasyclados,* and *S. dasyclados x viminalis*

**Fig. 5:**
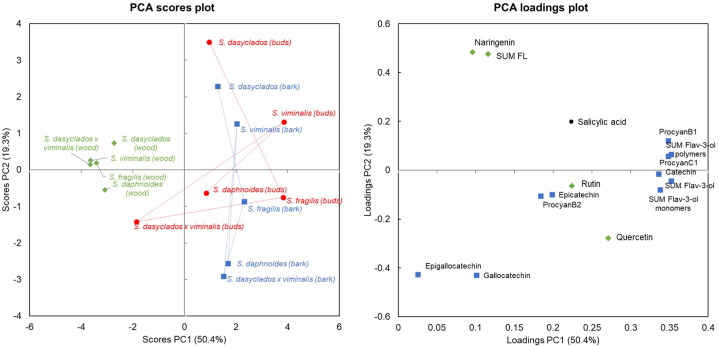
Principal component analysis of PCs measured in buds (red-filled circles), bark (blue-filled squares) and wood (green-filled rhombus) from five dormant varieties of willow. The left panel represents the PCA scores plot (sample distribution and variation); the right panel represents the PCA loadings plot (variables contributing to the data variation).

An overview of all measured PCs in *S. daphnoides, S. fragilis, S. viminalis, S. dasyclados,* and *S. dasyclados x viminalis* is shown in Fig. 5. PCA is a classical chemometric analysis method used for data dimensionality reduction by transforming it into a new coordinate system [33]. In this new system, the majority of the variation can be effectively represented within the first two dimensions, which are known as the principal components and helps with the clustering of samples based on similar responses [33]. The objects of the PCA are represented in the data matrix by wood, bark, and bud samples of five Salix species, while the LC-MS/MS quantified target compounds represent the variables of the PCA.

The principal components (PC) are selected as the first PC (PC1) accounts for most of the variation in the data set, while the second PC (PC2) accounts for the second largest variation in the dataset. In this data set, PC1 and PC2 describe 69.7 % of the total variance in the initial data matrix (Fig. 5). PC1 describes most of the variability (50.4 %), which correlates to the detected amounts of catechin, procyanidin B1, procyanidin C1 and the sum of total flavan-3-ols (as monomers and polymers). PC2 is defined by the high levels of naringenin and the total sum of flavonoids for some specific species and higher levels of epigallocatechin, gallocatechin, and quercetin for others. PC1 allowed for the distinction of two major groups in the dataset, the first consisting of the wood samples of *S. daphnoides, S. fragilis, S. viminalis, S. dasyclados,* and *S. dasyclados x viminalis* (Fig. 5). The second group consisted of the bark and buds samples of all Salix species, which were characterized primarily by the high contents of catechin, procyanidin B1, procyanidin C1 and the sum the sum of total flavan-3-ols (as monomers and polymers), with buds, particularly from S. viminalis and S. fragilis containing high levels of these compounds. In the direction of PC2, *S. dasyclados* and *S. viminalis* were separated from the rest of the samples through their high levels of naringenin and the total sum of flavonoids measured in bark and buds. In the opposite direction, bark from *S. daphnoides* and *S. dasyclados x viminalis* showed a closed grouping due to higher levels of epigallocatechin and gallocatechin (Fig. 5). Similar differences between bark and leaves studied in other Salix species have been recently reported, with particular discrepancies between levels of epicatechin, naringenin and rutin [34].

## 3 Conclusion

We have observed high variations in the concentrations of individual flavonoids, which were not species-specific except for naringenin. There was also a high fluctuation between buds and bark in the concentrations of the individual flavonoids, salicylic acid and TPC. However, constantly low concentration of all measured flavonoids, salicylic acid and TPC has been observed for wood. A higher concentration of polyphenolic compounds in the bark of willow is primarily a result of the bark's vulnerability to external threats and as a result the strong defense mechanism of willow against pathogens, herbivores, or other threats. The diverse willow tree species offer a rich source of high-value phenolic compounds with varied distribution among plant parts and different species, particularly emphasizing the bark's role as a robust defense mechanism against external threats. Kaempferol was only detected in buds. The exclusive presence of kaempferol in buds, but not in other parts of willow, suggests that different plant parts may serve specific ecological or defensive functions. Understanding the selective distribution of compounds like kaempferol can inform ecological and evolutionary studies. The highest concentrations were measured for catechin, naringenin and salicylic acid. *Salix dasyclados* was significantly higher in naringenin and salicylic acid (except for buds). Willow species with higher naringenin concentrations could hold economic significance. Naringenin is known for its potential health benefits and is used in the nutraceutical and food supplement industries. Therefore, willow species rich in naringenin might be valuable for the production of dietary supplements and functional foods, potentially contributing to economic opportunities. No other species can be assigned to produce specific flavonoids. In general, all five species of willow produced flavonoids and salicylic acid measured in this study. There was good agreement between LC-MS and Folin-Ciocalteu assay showing a similar trend of lower content of flavonoids and TPC in wood compared to bark and buds. This alignment validates the accuracy and reliability of the analytical methods used in the study and strengthens the overall credibility of the research findings. The highest TPC has been measured in the bark of *S. dasyclados x viminalis,* reaching 6.7 %, but at the same time, lowest in buds, 3 % for the same species. In general, all five varieties contained high TPC in buds and bark. The variation among different willow species and parts provides a wealth of opportunities for industries ranging from pharmaceuticals and nutraceuticals to biorefinery and environmental applications. The strategic selection of species and plant parts can optimize resource utilization and enhance the sustainability of various sectors within the circular bioeconomy. The potential for optimizing willow cultivation and product development based on phenolic composition is substantial and warrants further exploration.

## 4 Experimental

### 4.1 Willow collection and sample preservation

**Fig. 6:**
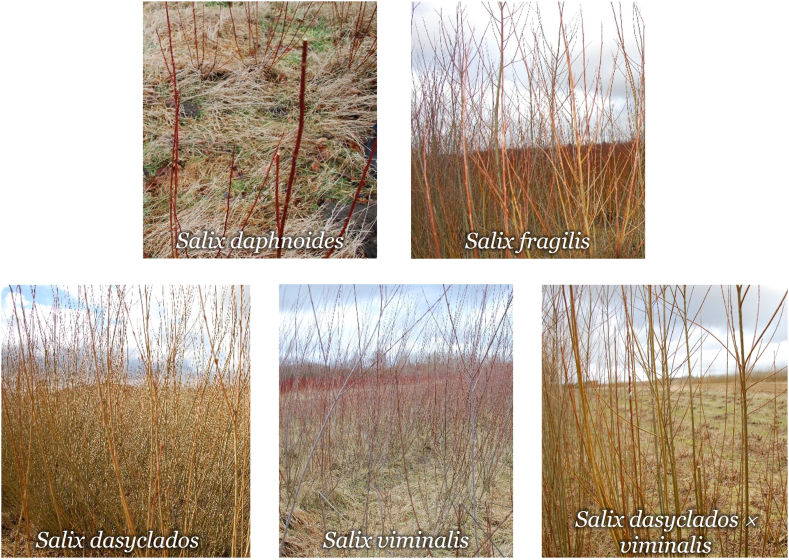
Field collection of five different varieties of willow.

**Fig. 7:**
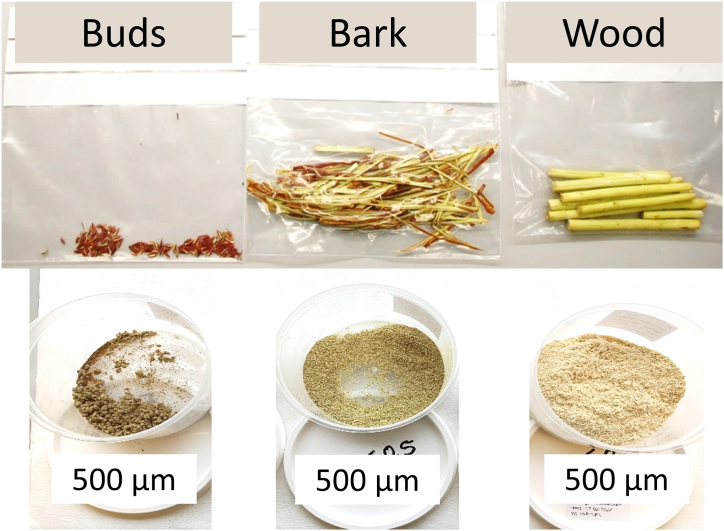
Sample preparation of buds, bark, and wood from different willow varieties.

**Fig. 8:**
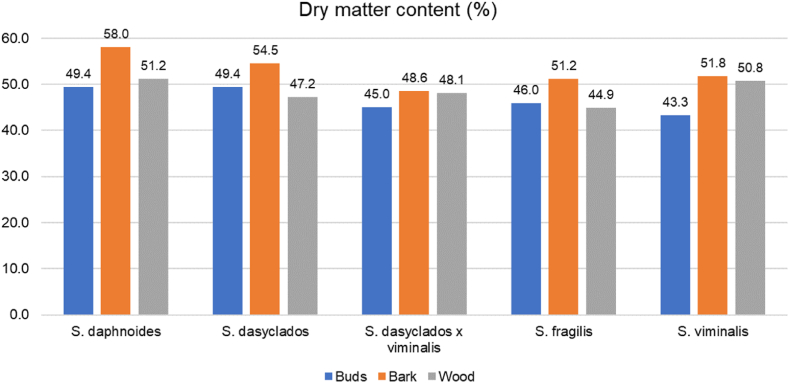
Dry matter content of *Salix daphnoides, Salix dasyclados, Salix dasyclados x viminalis, Salix fragilis,* and *Salix viminalis*.

Five varieties of organic winter dormant 3-year-old willow trees: *Salix daphnoides, Salix fragilis, Salix viminalis, Salix dasyclados* and *Salix dasyclados x viminalis* were selected for collection on February 17th, 2022, at Ny Vraa Biorefinery in Northern Jutland, Denmark (Fig. 6). Eight to ten branches were manually cut using a bypass pruner and preserved in plastic bags during 1 h of transportation to Aarhus University, Campus Viborg-Foulum, Denmark. The branches were randomly cut from different willow shrubs, two cuttings were taken from four to five individual shrubs to get a better representation of each variety field. Moreover, each individual shrub was selected to be distanced approximatively 5 m from the previous sampled shrub. The buds were peeled off in the laboratory, and branches were sorted based on their diameter. The branches with a diameter larger than 5 mm were cut into 10 cm pieces, and the bark was separated from the wood. The buds, bark and wood were stored in plastic bags at −20 °C until further processing (Fig. 7). To avoid further metabolic changes, the samples were freeze-dried after collection. Before freeze-drying, buds, bark and wood were placed at −80 °C for 24 h. The freeze-dryer ScanVac CoolSafe (LaboGene A/S Lillerød, Denmark) operated at −40 °C for 72 h. After freeze-drying, the samples were placed into an exicator for 10 min before the final weighing. Dry matter content varied between 42 and 58 % (Fig. 8). Milling of the buds, bark and wood samples was performed in the IKA TUBE-MILL 100 Control mill (Thermo Fisher Scientific, Waltham, MA, U.S.A; Merck KGaA, Darmstadt, Germany) using MT-40.100 disposable grinding chambers (IKA®-Werke GmbH & Co. KG, Staufen, Germany) at 25000 rpm for app. 2 min. The homogenous samples were further screened through a 500 μm sieve (Buch & Holm A/S, Herlev, Denmark), Fig. 2. The buds, bark and wood samples were stored at −20 °C before further analyses.

### 4.2 Standards and chemicals

The following standards were purchased from Sigma-Aldrich (Merck KGaA, Darmstadt, Germany): catechin, epicatechin, gallocatechin, epigallocatechin, catechin gallate, gallocatechin gallate, procyanidin B1 and B2, procyanidin A2, procyanidin C1, kaempferol, quercetin, rutin, salicylic acid, catechin-2,3,4–^13^C_3_ 99 atom % ^13^C (98 % CP), gallocatechin-2,3,4–^13^C_3_ ≥ 99 atom % ^13^C (≥97 % CP), catechin-2,3,4–^13^C_3_ gallate ≥99 atom % ^13^C (≥97 % CP), salicylic acid-D_4_ certified reference material. Naringenin was purchased from Thermo Scientific (Waltham, MA, U.S.A).

The following chemicals were purchased from Sigma-Aldrich (Merck-Millipore, Merck KGaA, Darmstadt, Germany): dimethyl sulfoxide (DMSO), hydrochloric acid (HCl, 37 %), formic acid (FA, LiChropur 98–100 % LCMS grade), 2 M Folin-Ciocalteu′s phenol reagent (47641-500 ML-F), gallic acid (G7384-100G) and β-glucuronidase type H-1 from *Helix pomatia*. Sodium bicarbonate (S6014-500G, Fluka), methanol (85681.320, VWR), and acetonitrile (ACN, HiPerSolv Chromanorm) were purchased from VWR Chemicals (Radnor, PA, U.S.A.). Sodium acetate was obtained from Merck (Darmstadt, Germany) and glacial acetic acid from Fluka/Sigma-Aldrich (Thermo Fisher Scientific, Waltham, MA, U.S.A; Merck KGaA, Darmstadt, Germany).

### 4.3 Buds, bark, and wood extraction

The extraction for total polyphenol content (TPC) and Liquid Chromatography-Mass Spectrometry (LC-MS/MS) of buds, bark, and wood was performed with 100 % MeOH + 1 % HCl according to Curtasu et al. (2023) [35]. Briefly, 50 mg willow material was extracted with 2 mL solvent at room temperature, and the samples were shaken for 1 h and centrifuged at 1962 rcf for 10 min at 20 °C. The supernatant was transferred to a new tube and stored at −80 °C until further analysis. Before LC-MS/MS analyses, extracted bark samples (25 μL of each extract) were diluted 100-fold in a working solvent of 5 % ACN (v/v) and 1 % FA (v/v) in water containing a mixture of internal standards (IS mix), reaching the final concentration of the standard curve. The IS mix contained labelled standards: catechin ^13^C_3_, gallocatechin ^13^C_3_ and catechin ^13^C_3_ gallate dissolved in DMSO at 1 mg/mL, salicylic acid-D_4_ dissolved in acetonitrile in concentration 100 μg/mL, and ^13^C_3_ enterolactone was dissolved in acetonitrile in a concentration of 1 mg/mL as described by Curtasu et al. (2023) [35]. As temperature, time of extraction and different solvents can affect compound stability Supplementary Fig. S1 and Fig. S2 show the effects of increased temperature and extraction time on the stability of rutin and quercetin, respectively. Compound-dependent parameters optimized by syringe infusion of pure standards, ions and masses selected for quantification of compounds, declustering potential (DP), collision energy (CE), and cell exit potential (CEP) have all been previously described in the supplementary materials section of Curtasu et al. (2023) [35]. The extractions were performed in triplicates for bark and wood and only in duplicates for buds due to a lack of sufficient material. Further hydrolysis of the extracts was performed to liberate salicylic acid aglycon following the protocol described by Curtasu et al. (2023) [35].

### 4.4 Total polyphenol content (TPC) assay using Folin-Ciocalteu

The Folin-Ciocalteu assay was performed on a 2103 EnVision Multilabel Reader (PerkinElmer Life and Analytical Sciences, Shelton, CT, U.S.A) in 96-well plates (Nunc MicroWell, Thermo Fisher Scientific, Waltham, MA, U.S.A) and the absorbance was measured at 630 nm. The assay was performed according to Hong et al. (2020) [36]. Extracts of buds, bark, and wood (10 μL) were mixed with 25 μL of 1 M Folin-Ciocalteu reagent (1:1 Folin-Ciocalteu 2 M:MilliQ), 25 μL 20 % sodium bicarbonate and 150 μL MilliQ using a multichannel pipette. The plate was incubated for 30 min at room temperature before reading. Gallic acid has been used to prepare standard curves in the range of 31.25, 62.5, 125, 250, 500, 750, and 1000 μg/ml. Total polyphenols concentration was calculated according to the gallic acid standard curve and expressed as mg equivalents of gallic acid/g of dry matter.

### 4.5 Liquid Chromatography-Mass Spectrometry

LC-MS/MS was performed according to Curtasu et al.(2023) [35]. Briefly, extracted buds, bark and wood were diluted 100-fold with 5 % ACN containing internal standard mix (labelled compounds) and analyzed on a microLC 200 series from Eksigent/AB Sciex (Redwood City, CA, USA) coupled to a QTrap 5500 mass spectrometer from AB Sciex (Framingham, MA, USA). Compounds were separated on Kinetex 1.7 μm Phenyl-Hexyl, 100–2.1 mm column using mobile phases consisting of solvent A (1 % FA in MilliQ) and solvent B (0.1 % FA in ACN) and gradient from 10 % to 90 % of solvent B during 10 min. The mass spectrometer was operated in negative ionization mode using multiple reaction monitoring (MRM) scanning and electrospray ionization (ESI). Compounds were quantified based on standard curves prepared from their authentic standards containing both labelled and non-labelled compounds. The data analysis was performed in the Analyst software 1.7.1 from AB Sciex (Framingham, MA, USA). Sample preparation procedures can lead to the loss of analytes through clean-up and up-concentration when the sample matrix is very complex. Therefore, recovery and matrix effects of the analysis were previously assessed during method development which are described by Curtasu et al. (2023) [35]. Briefly, spiking of compound at low, medium, and high levels resulted in a high recovery percentage for all analytes tested demonstrating a good robustness of the extraction and analysis procedure. Recoveries for most PCs were close to 100 % [35].

### 4.6 Statistics and calculations

The measured concentrations of compounds by LC-MS/MS were used to calculate the final concentrations by accounting for the extraction, dilution factors, and the weight of the extracted material.

CPC= ((Cmeasured x Ve x DF)/Wdry buds-bark-wood)/1000000

where CPC is the final concentration of the PC (mg/g), Cmeasured is the measured concentration (ng/mL), Ve is the extraction volume (mL), DF is the dilution factor, and Wdry buds-bark-wood is the weight of dry buds, bark and wood portion used for the extraction. The final values were reported on a dry matter (DM) basis (mg/g). Further, the average and standard deviation of the three extractions were calculated.

The statistical analyses were conducted in SAS 9.4 (SAS Institute Inc., Cary, NC, USA) using the GLM procedure. The differences between willow varieties were analyzed using the following linear model:

Yij = μ + αi + εij,

where Yij is the dependent variable, μ is the overall mean, αi is the fixed effect of willow variety (i = *S. daphnoides, S. fragilis, S. viminalis, S. dasyclados, and S. dasyclados x viminalis*), and εij is the residual error component. Least squares mean estimates are reported. Significance was declared at P ≤ 0.05 and trend at 0.05 < P ≤ 0.10.

Chemometric analysis using principal component analysis was performed in LatentiX 2.13 (Latent5 Aps, Denmark), where the data set was imported and scaled using the autoscale function, where all variables become equally important, and the variables are compared based on correlations. Observation points are represented by the samples of buds, bark and wood of five willow species (*S. daphnoides, S. fragilis, S. viminalis, S. dasyclados, and S. dasyclados x viminalis), while the variables are represented by the LC-MS/MS concentrations of PCs.*

## Funding

The analysis conducted in this study is part of the ECOCO2W project (Tannins of willow and hemp as organic feed additives for methane reduction in dairy cows). The project ECOCO2W is part of the Organic RDD 7 program, which is coordinated by the International Center for Research in Organic Food Systems (ICROFS). It has received grants from the Green Growth and Development program (GUDP) under the Danish Ministry of Food, Agriculture, and Fisheries.

## Data availability statement

Has data associated with your study been deposited into a publicly available repository? Yes.

Raw data from: Quantitative distribution of flavan-3-ols, flavonols, flavanone and salicylic acid in five varieties of winter dormant Salix spp. by LC-MS/MS (view at https://dataverse.harvard.edu/dataset.xhtml?persistentId=doi:10.7910/DVN/N9GFW2) was published in Harvard Dataverse (view at https://dataverse.harvard.edu/dataverse/harvard).

## Ethics statement

Review and/or approval by an ethics committee was not needed for this study because through the nature of this study no animal subjects, live vertebrates or higher invertebrates were used. Informed consent was not required for this study because the study did not involve participants/patients.

## CRediT authorship contribution statement

**Mihai Victor Curtasu:** Writing – review & editing, Writing – original draft, Visualization, Validation, Methodology, Investigation, Formal analysis, Data curation. **Natalja P. Nørskov:** Writing – original draft, Validation, Resources, Project administration, Methodology, Investigation, Funding acquisition, Conceptualization.

## Declaration of competing interest

The authors declare that they have no known competing financial interests or personal relationships that could have appeared to influence the work reported in this paper.
