# Supplementary material for: Quantitative distribution of flavan-3-ols, procyanidins, flavonols, flavanone and salicylic acid in five varieties of organic winter dormant Salix spp. by LC-MS/MS
Source: Heliyon. 2024 Feb 1;10(3):e25129. doi: 10.1016/j.heliyon.2024.e25129 (PMC10850552; doi:10.1016/j.heliyon.2024.e25129)
Supplement: Multimedia component 1 [file mmc1.docx]

*Supplementary Materials Section*

Quantitative distribution of flavan-3-ols, flavonols, flavanone and salicylic acid in five varieties of winter dormant *Salix spp*. by LC-MS/MS

Mihai Victor Curtasu and Natalja P. Nørskov

Department of Animal and Veterinary Sciences, Aarhus University, Blichers Alle 20, 8830 Tjele, Denmark

Corresponding author: Mihai-Victor Curtasu

Phone: +45 8715 0000

E-mail: mihai.curtasu@anivet.au.dk

Present address: AU-Campus Viborg, Aarhus University, Blichers Alle 20, 8830 Tjele, Denmark

Supplementary Materials Section


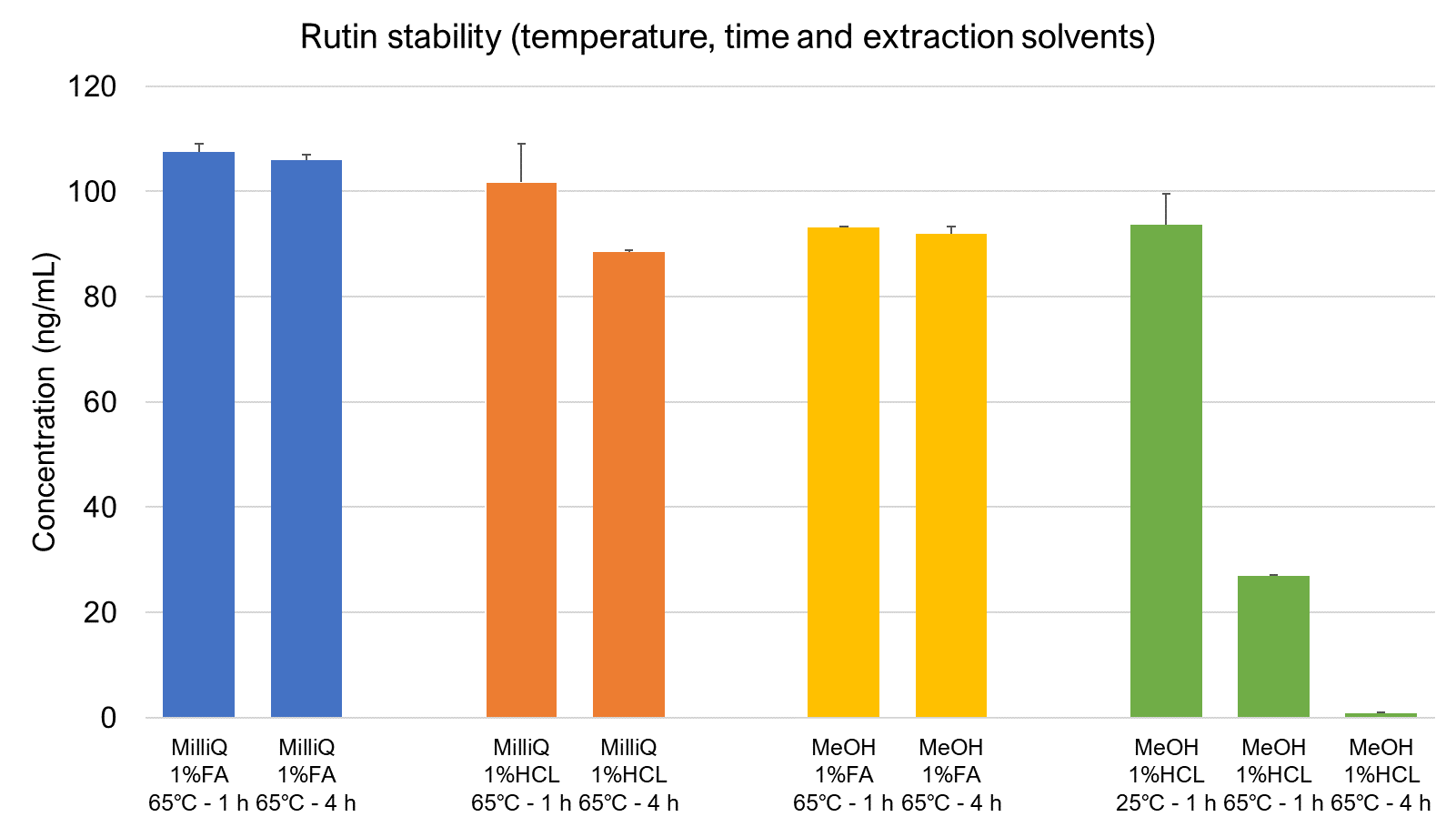


Figure S1. Rutin compound stability. Measurement of the effect of extraction temperature, time of extraction, and different extraction solvents, performed in bark from willow (Salix daphnoides). Values presented are averages of two duplicate analysis and error bars indicate S.E.


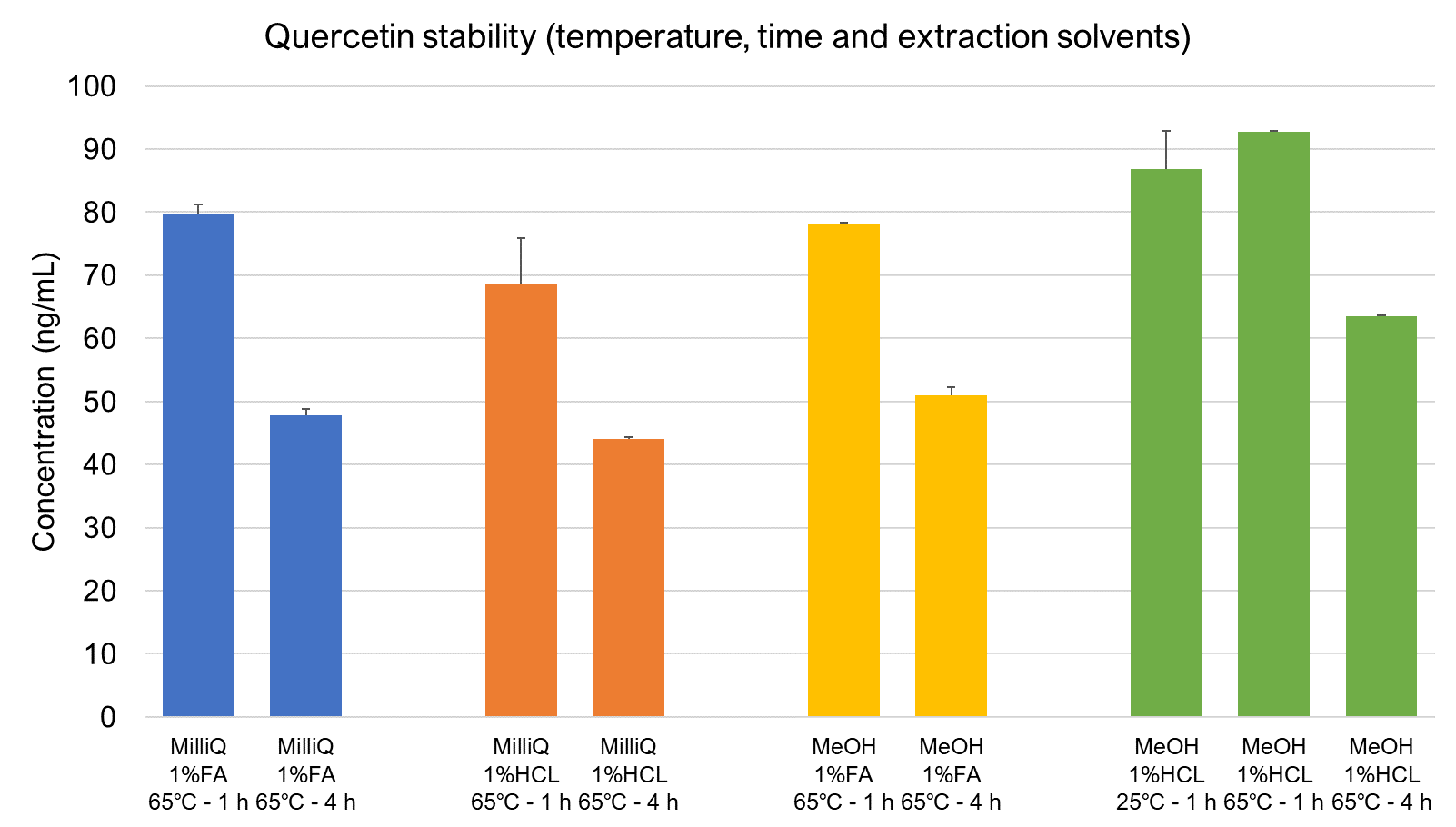


Figure S1. Quercetin compound stability. Measurement of the effect of extraction temperature, time of extraction, and different extraction solvents, performed in bark from willow (Salix daphnoides). Values presented are averages of two duplicate analysis and error bars indicate S.E.
